# Analysis of Factors Influencing Telephone Call Response Rate in an Epidemiological Study

**DOI:** 10.1155/2014/179375

**Published:** 2014-10-21

**Authors:** Jorge Matías-Guiu, Pedro Jesús Serrano-Castro, José Ángel Mauri-Llerda, Francisco José Hernández-Ramos, Juan Carlos Sánchez-Alvarez, Marisa Sanz

**Affiliations:** ^1^Department of Neurology, Hospital Clínico San Carlos, Avenida Prof. Martín Lagos S/N, 28040 Madrid, Spain; ^2^Neurology and Neurophysiology Unit, Complejo Hospitalario Torrecárdenas, 04009 Almería, Spain; ^3^Department of Neurology, Hospital Clínico Universitario Lozano Blesa, 50009 Zaragoza, Spain; ^4^Neurology Unit, Complejo Hospitalario Llerena-Zafra, 06900 Badajoz, Spain; ^5^Department of Neurology, Hospital Clínico Universitario San Cecilio, 18012 Granada, Spain; ^6^Research Operations Office, IT Department, Spanish Society of Neurology, San Sebastian de los Reyes, 28701 Madrid, Spain

## Abstract

Descriptive epidemiology research involves collecting data from large numbers of subjects. Obtaining these data requires approaches designed to achieve maximum participation or response rates among respondents possessing the desired information. We analyze participation and response rates in a population-based epidemiological study though a telephone survey and identify factors implicated in consenting to participate. Rates found exceeded those reported in the literature and they were higher for afternoon calls than for morning calls. Women and subjects older than 40 years were the most likely to answer the telephone. The study identified geographical differences, with higher RRs in districts in southern Spain that are not considered urbanized. This information may be helpful for designing more efficient community epidemiology projects.

## 1. Introduction

Descriptive epidemiology research involves collecting data from broad geographical areas and large numbers of subjects. Obtaining these data requires approaches designed to achieve maximum participation or response rates (RRs) among respondents possessing the desired information. With this in mind, researchers conducting community studies have debated the use of different strategies, whether face-to-face, mail based, or web based [1]. Potential methods for collecting specific information [2] range from door-to-door studies, used in research on low-prevalence diseases, to those conducted using traditional mail, telephone [3], the Internet [4, 5], interactive voice response (IVR) [6, 7], computer-assisted telephone interview (CATI) [8, 9], or combinations of the above methods [10–13]. In any case, the method delivering the highest RR would be considered the most efficient and appropriate.

The presence of a low RR is an important issue to consider in field survey design because it may introduce a significant bias in the evaluation of results. Response rates have decreased from the 1970s to the first decade of the new millennium due to apparently circumstantial reasons [14]. Although biases of this type are considered to hold little relevance for prevalence estimations [15], achieving a high RR should be a priority for researchers designing epidemiological studies because the decisions made in answer to nonresponses may affect the results [16]. Furthermore, low RRs give rise to longer study durations and thus to cost increases, a situation which contributes to lower management efficiency in research.

EPIBERIA is a population-based epidemiological study of the prevalence of epilepsy in subjects aged over 18 which makes use of data collected by telephone from three representative Spanish regions [17]. The objective of the present study is to analyze RR in a population-based epidemiological study and to identify factors implicated in consenting to participate in community studies; this information may be helpful for designing more efficient community epidemiology projects.

## 2. Material and Methods

### 2.1. Description of the EPIBERIA Study

EPIBERIA is a population-based epidemiological study of a population sample that is representative of Spain as a whole, drawing from an independent census provided by databases of healthcare system users in three Spanish regions (data as of January 1, 2012). Its main objective is to measure the lifetime prevalence of epilepsy in subjects over 18 years old. The study designed to fulfill the established objectives was divided into two phases: screening (phase 1) and confirmation (phase 2). The screening phase consisted of the random administration of the EPIBERIA questionnaire by telephone to a population sample extracted from the three regions selected as representative of Spain as a whole. In the confirmation phase, participants selected in the screening phase were invited to attend a face-to-face interview with a neurologist with experience in diagnosing epilepsy. The design, methods, and results of the EPIBERIA study have been described elsewhere [17].

### 2.2. Geographical Regions under Study

The study was carried out in three Spanish regions with different demographic characteristics. (a) Health District III in Zaragoza, with a population of 168 378 inhabitants over 18 years old, spans a predominantly urbanized area with an average annual rainfall of 315 mm. (b) Torrecárdenas Primary Care District in Almeria is a mixed urban and rural region (32% of people are considered rural) with a population of 236 177 inhabitants over 18 years old and an average annual precipitation of 196 mm. (c) The Sevilla Norte Health District, which includes the Vega del Guadalquivir and Sierra Norte areas, is mostly rural with a total population of 243 461 inhabitants over 18 years old and an average annual rainfall of 810 mm.  Table 1 displays the distribution of the participants by age group and region.

### 2.3. Sample Selection

Researchers used randomization software to select the sample from independent census data kept in healthcare system user databases maintained by public healthcare institutions in the regions of Andalusia (containing the Almeria and Seville healthcare districts) and Aragon (containing the Zaragoza healthcare district). Since the Spanish National Health System provides universal coverage, these databases include almost the entire census population. IT services from the selected health districts extracted a list of 3000 random individuals from each district's user database. Telephone numbers for a total of 9000 individuals were therefore provided to the EPIBERIA researchers.

### 2.4. Methods

Random selection was used to extract a sample of 3000 individuals aged 18 and older from each database representing a selected geographical area. From each of these three geographical pools, 600 subjects were then randomly selected to participate in the survey. Assuming an RR of about 20% (one in five phone calls would deliver a participant), we also selected 2400 substitutes in case selected individuals did not respond. Substitutes were chosen using the same randomization process and matched to one of the selected individuals (4 substitutes per 1 selected subject) before the telephone calls were made.

### 2.5. Telephone Survey

The telephone survey was carried out by interviewers trained for that purpose. None of them were medical personnel or had prior knowledge of epilepsy. Interviewers were permitted to clarify questions as long as they did not provide the participant's responses. The interview lasted a maximum of 15–20 minutes; in this time, interviewers presented a brief introduction of the study, requested the subject's participation, and, in suspected cases of epilepsy, suggested that the subject be reexamined by an experienced neurologist. All subjects verbally gave informed consent to participate in the study. Interviewers called up to three different times to reach the selected candidate. If that person could not be contacted or refused to participate in the study, the interviewer called a substitute. While making the phone call, the interviewer entered responses to the questionnaire in an electronic database. When finished, interviewers printed out and filed the completed questionnaire.

### 2.6. Mass Media Campaign

A mass media campaign was carried out in all three districts during the weeks preceding the EPIBERIA screening phase. This campaign was carried out by the Spanish Society of Neurology's press office which sent a press release to all local media outlets (21 in Zaragoza, 10 in Almeria, and 34 in Seville).

### 2.7. Ethical Considerations

All procedures were performed in accordance with guidelines established by the Declaration of Helsinki and by Spanish law [18]. The protocol was evaluated and approved by the ethics committee for each region. Participants' personal data were rendered anonymous with a code and encrypted to limit access to EPIBERIA researchers and personnel at the Spanish Society of Neurology's Department of IT and Data Management. Researchers were only granted access to the data pertinent to their roles in the study. The lead researcher from EPIBERIA had access to the database linking the anonymous codes to each participant. Data files were designed according to criteria established by Spanish data protection laws.

### 2.8. Data Included in the Present Analysis

The analysis described here examined information included in the screening phase (phase 1) database from the EPIBERIA study. These data include information on subjects included in the calling list and their responses.

### 2.9. Analyzed Variables

Analyzed variables were as follows:

- contact rate (CR), calculated as the percentage of answered telephone calls out of the total number of calls; calls classified as unanswered included both failure to make contact and wrong numbers (such cases were infrequent);
- response rate for answered calls (RR), calculated as the number of calls in which the subject consented to participate in the study as a percentage of the total answered calls;
- response rate for total calls (RRt), calculated as the number of calls in which the subject consented to participate in the study as a percentage of the total phone calls; these rates were broken down by time of call (morning or afternoon) and the participant's district, sex, and age group.

Data describing the mass media campaign in each district was expressed as follows: (a) total number of appearances (TNA) in media outlets; (b) media appearances out of outlets contacted, calculated as appearances divided by the total number of media outlets that received the press release; (c) intensity of media appearance, calculated by assigning 1 point for an appearance in written press, 2 for radio, and 3 for television, plus 3 additional points if one of the researchers was interviewed by a media outlet, and dividing the total by the number of media outlets that received the press release.

### 2.10. Statistical Analysis

Data were expressed as percentages and the corresponding 95% confidence intervals (95% CI) were calculated. Figures were drawn using Epidat 4.0 software.

## 3. Results

### 3.1. Phone Call Answer Rate

**Figure 1:**
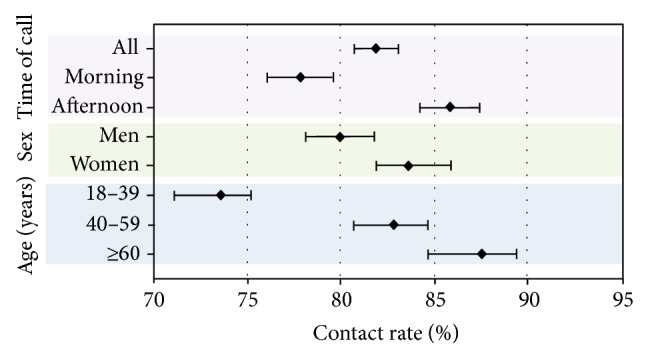
Contact rate broken down by time of call, sex, and age.

Of the total of 3876 telephone calls, 3175 were answered and 701 were not, resulting in a CR of 81.9% (95% CI, 80.7–83.1). Broken down by region, CR in Zaragoza was 75.95% (95% CI, 73.9–77.9, with 416 unanswered calls out of 1729); in Almeria, 87.56% (95% CI, 85.4–89.4, with 129 unanswered calls out of 1038); and in Seville, 85.93% (95% CI, 83.8–87.9, with 156 unanswered calls out of 1109). Significant differences were found between Zaragoza and the other two regions. Regarding time of call, CR was 77.88% in the morning (95% CI, 76.1–79.6, with 470 unanswered calls out of 2125) and 85.80% in the afternoon (95% CI, 84.2–87.4, with 231 unanswered calls out of 1751). Therefore, calling was significantly more effective during afternoon hours than during morning hours. In the breakdown by sex, CR was 79.98% for men (95% CI, 78.1–81.8, with 367 unanswered calls out of 1834) and 83.61% for women (95% CI, 81.9–85.9, with 334 unanswered calls out of 2038). Calls were answered more frequently by women than by men. In the breakdown by age, subjects aged 18–39 had a CR of 73.61% (95% CI, 71.1–75.2, with 487 unanswered calls out of 1819); when also broken down by sex, the rate for men was 67.42% (95% CI, 64.4–70.3, with 314 out of 964) and for women was 79.76% (95% CI, 84.3–88.9, with 173 out of 855) (Figure 1). In subjects aged 40 to 59, CR was 82.80% (95% CI, 80.7–84.7, with 237 unanswered calls out of 1378); broken down by sex, the rate was 81.45% for men (95% CI, 78.3–84.2, with 122 out of 658) and 84.02% for women (95% CI, 81.2–86.5, with 115 out of 720). CR in subjects older than 60 was 87.16% (95% CI, 84.6–89.4, with 96 unanswered calls out of 748); in this age group, the rate was 84.12% for men (95% CI, 79.7–87.8, with 50 out of 315) and 89.37% for women (95% CI, 84.6–92.0, with 46 out of 433). CR in participants aged 18 to 39 was significantly lower than in other age groups. Although that rate was higher among subjects aged over 60 than in the group aged 40 to 59 years, the difference was not statistically significant. CR was higher among females in all three age groups, but the difference was only significant for the group aged 18–39 years.

### 3.2. Response Rate for Answered Calls

**Figure 2:**
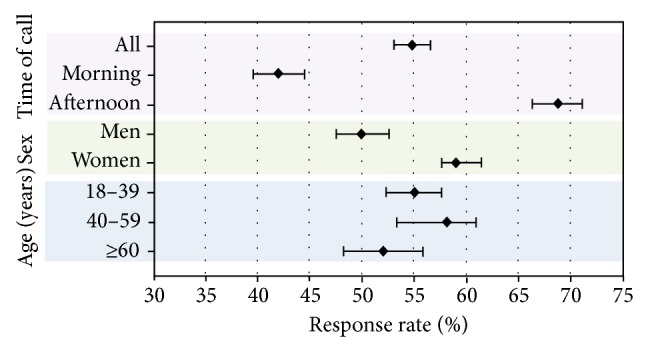
Response rate broken down by time of call, sex, and age.

**Figure 3:**
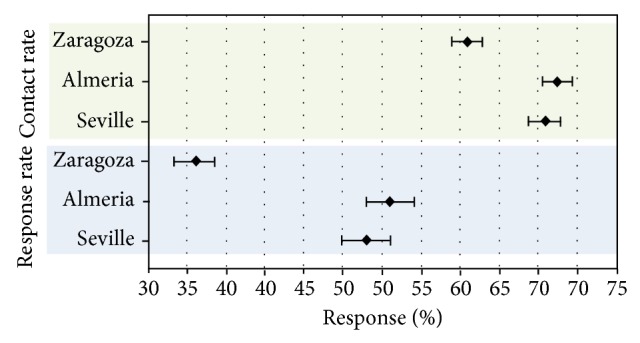
Contact rate and response  rate by region.

Of the 3175 individuals who answered the phone call, 1741 agreed to participate in the study and 1434 declined, resulting in a response rate (RR) of 54.83% (95% CI, 53.1–56.6). Broken down by region, RR was 41.23% in Zaragoza (95% CI, 38.3–43.6, with 541 favorable responses out of 1313 completed calls); 66.01% in Almeria (95% CI, 62.9–69.0, 600 out of 909); and 62.95% in Seville (95% CI, 59.8–66.0, 600 out of 953). RR was significantly lower in Zaragoza than in Almeria or Seville (Figure 3). Regarding time of the call, RR was 41.99% in the morning (95% CI, 39.6–44.5, with 695 favorable responses out of 1655 calls) and 68.81% in the afternoon (95% CI, 66.4–71.1, with 1046 favorable responses out of 1520 calls). Response rates were therefore significantly higher for afternoon calls than for morning calls. Broken down by sex, RR was 50.03% in men (95% CI, 47.5–52.6, with 734 favorable responses out of 1467 calls) and 59.09% in women (95% CI, 56.7–61.4, with 1007 favorable responses out of a total of 1704 calls), indicating that calling women was more effective than calling men. Broken down by age, RR in subjects aged 18–39 was 54.95% (95% CI, 52.3–57.6, with 732 favorable responses out of 1332 calls) (Figure 2). Within that age group, RR was 50.61% in men (95% CI, 46.8–54.4, 329 out of 650) and 59.09% in women (95% CI, 55.4–62.7, 403 out of 682). In subjects aged 40–59, RR was 58.19% (95% CI, 53.3–61.0, with 664 favorable responses out of 1141 calls) and 49.09% in men (95% CI, 44.9–53.3, 263 out of 536) and 66.28% in women (95% CI, 62.4–69.9, 401 out of 605). RR was 51.99% in subjects older than 60 (95% CI, 48.2–55.8, with 339 favorable responses out of 652 calls) or 53.58% in men (95% CI, 47.6–59.5, 142 out of 265) and 50.90% in women (95% CI, 46.1–56.0, 197 out of 387). Despite rates being lower in subjects aged over 60, age-related differences were not significant. RR was significantly higher in women for both the 18–39 and the 40–59 age groups, but there were no significant sex differences in the group older than 60.

### 3.3. Response Rate for Total Calls

The response rate for total calls (RRt) was 44.91% (95% CI, 43.4–46.5) with 1741 favorable responses to a total of 3876 calls. Broken down by region, RRt was 31.28% in Zaragoza (95% CI, 29.1–33.5, with 541 out of 1729 calls); in Almeria, 57.80% (95% CI, 54.6–60.8, 600 out of 1038); and in Seville, 54.10% (95% CI, 51.2–57.0, 600 out of 1109). RRt was significantly lower in Zaragoza than in the other two regions. While RRt was higher in Almeria than in Seville, the difference is not statistically significant. Regarding calling times, RRt was 32.70% for morning calls (95% CI, 30.7–34.7, with 695 favorable responses out of 2125 calls), and 59.73% for afternoon calls (95% CI, 57.4–62.0, with 1046 favorable responses out of 1751 calls). Calling was therefore significantly more effective in the afternoon than in the morning. Broken down by sex, RRt was 40.02% in men (95% CI, 37.8–42.3, 734 out of 1834) and 49.41% in women (95% CI, 47.2–51.6, 1007 out of 2038). Therefore, calling women was more effective than calling men. Broken down by age, RRt in the 18–39 age group was 40.24% (95% CI, 38.0–42.5, with 732 favorable responses out of 1819 calls); when also broken down by sex, RRt was 34.10% in men (95% CI, 31.2–37.2, 329 out of 964) and in women, 47.10% (95% CI, 43.8–50.0, 403 out of 855). RRt in the 40–59 age group was 48.18% (95% CI, 45.6–50.8, with 664 out of 1378 calls), specifically, 39.96% in men (95% CI, 36.3–43.8, 263 out of 658) and 55.69% in women (95% CI, 52.0–59.3, 401 out of 720). RRt in the age group over 60 was 45.32% (95% CI, 41.8–48.9, with 339 out of 748 calls), specifically, 45.07% in men (95% CI, 39.7–50.6, 142 out of 315) and 45.49% in women (95% CI, 40.9–50.2, 197 out of 433). RRt was significantly lower in individuals aged 18–39 than in the other age groups. Although RRt was higher in subjects over 60 than in those aged 40–59, the difference was not significant. RRt in the 18–39 and 40–59 age groups was significantly higher in women than in men but displayed no significant differences in subjects over 60.

### 3.4. Intensity of the Media Campaign

The campaign resulted in 59 appearances in media outlets, 21 in Zaragoza (out of 21 possible media outlets, yielding an appearance rate per outlet = 1.0), 15 in Almeria (rate per outlet = 1.5), and 23 in Seville (rate per outlet = 0.67). All media appearances in Zaragoza and Seville were in written press only, whereas local television programs were also presented in Almeria. Researchers were interviewed by media outlets in Almeria and Zaragoza but not in Seville. The intensity of the mass media campaign in each region is shown in Table 2.

## 4. Discussion

Although there have been some randomized comparative studies on participation or response rate (RR), information from published literature mostly reflects a variety of other methods. While different studies have supported telephone [19], e-mail, IVR [20], or Internet-based surveying [21], the majority indicate that telephone-based studies achieve the highest response rates [22, 23], surpassing traditional mail [24]. Some studies indicate that a high prevalence of nonresponses may bias results [25, 26], but most authors consider that this characteristic does not affect the results of field surveys [27–29]. This may not be true, however, if the patients the study aims to identify have cognitive impairment [30], especially when there are specific reasons justifying nonresponse [31]. In light of the above, our study was designed to achieve an optimized RR using a structured telephone-based model in which a trained interviewer would present the questionnaire. Although the RR estimated during the design process was 20%, the observed RR was 44.91%, which is higher than rates reported in the literature [32]. Therefore, data from this study let us draw conclusions that may be instrumental in increasing the efficiency of future population-based epidemiological studies.

### 4.1. Contacting Candidates and Obtaining Consent to Participate

Contact with the candidates selected as a random sample was established in 81% of the phone calls. There were significant differences regarding time of the call, given that calls made in the afternoon were more likely to be answered. Women answered more frequently than men, and the contact rate was higher in older age groups. Subjects consented to participate in 54.83% of the answered calls and 44.90% of the total calls. Response rate was also higher for calls made during the afternoon, a finding that suggests that calling in the morning is associated not only with a lower probability of establishing contact, but also with candidates having less available time and being less inclined to cooperate. Response rates for morning and afternoon calls differed significantly at 41.99% and 68.81% respectively. Women consented to participate more frequently than men. No age effect was found for RR although the mean rate was lower in subjects over 60 years old. Individuals in this age group seem to answer calls more frequently although their RR is lower.

### 4.2. Comparison between Districts under Study

Districts under study displayed different phone call answer rates and response rates, with Zaragoza showing the lowest values for both CR and RR. For instance, RR in this region was 41.23% (95% CI, 38.3–43.6) versus 66.01% in Almeria (95% CI, 62.9–69.0) and 62.95% in Seville (95% CI, 59.8–66.0). The Zaragoza health district is considered predominantly urban whereas the other two regions are considered rural or mixed. Therefore, one way to interpret these results might be to posit that RR is lower in urban populations. Moreover, Zaragoza is located further north than the other regions. Since it is traditionally believed that people in southern Spain are more extroverted than those in the north, geographical personality patterns might also provide an explanation for this difference.

### 4.3. Impact of Mass Media on the Response Rate

A mass media campaign was carried out in the districts in question to raise local awareness about the study. Prior knowledge has been shown to have a significant effect on participation rate in telephone-based studies [33–35], especially in rural areas. While some researchers have indicated that providing background information, such as letters presenting the study [36, 37], may help increase RR [38–42], other studies do not support this hypothesis [43, 44]. Our study was unable to demonstrate a correlation between the number or intensity of appearances in media outlets and CR.

## 5. Conclusions

The analysis allowed us to characterize factors associated with the contact rates and participation rates in a telephone-based community epidemiological study. Rates exceeded those reported by the literature and they were higher for afternoon calls than for morning calls. Women and subjects older than 40 years were the most likely to answer the telephone. The study identified geographical differences, with higher RRs in districts in southern Spain that are not considered urbanized. A better understanding of factors influencing response rates will be very useful for designing more efficient and cost-effective epidemiological studies.

## Figures and Tables

**Table 1:** Distribution of inhabitants by region and age.

	18–39 years	40–59 years	≥60 years	Total
Almeria	99 617	81 403	55 157	236 177
Zaragoza	57 042	54 411	56 925	168 378
Seville	92 063	99 991	51 407	243 461
Total	**248 722**	**235 805**	**163 489**	**648 016**

**Table 2:** Intensity of the mass media campaign in each region.

	RR Mean (95% CI)	TNA	Appearance rate per media outlet	Intensity
Almeria	41.23% (38.3–43.6)	15	1.50	1.14
Zaragoza	66.01% (62.9–69.0)	21	1.00	2.00
Seville	62.95% (59.8–66.0)	23	0.67	0.67

RR = response rate; TNA = total number of appearances in mass media.
